# Recurrent fever, arthralgia and asymmetric genu varum of unexpected etiology

**DOI:** 10.1186/1546-0096-12-S1-P229

**Published:** 2014-09-17

**Authors:** Liliana Quaresma, Juan Gonçalves, Paula Estanqueiro, Manuel Salgado

**Affiliations:** 1 Centro Hospitalar do Baixo-Vouga - Aveiro, Aveiro, Portugal; 2 Hospital do Divino Espírito Santo, Ponta Delgada, Portugal; 3 Hospital Pediátrico de Coimbra, Coimbra, Portugal

## Introduction

Congenital syphilis is still a public health issue worldwide. Beyond the neonatal period, clinical findings may be subtle and nonspecific. We report an unusual case of congenital syphilis with recurrent fever, knee pain and severe anemia in a 28 month-old boy, despite standard penicillin treatment early in life.

Congenital syphilis should be considered throughout early childhood, especially if past or familiar medical story of syphilis.

## Objectives

Not applicable

## Methods

Not applicable

## Results

### Case Report

A 28-month-old boy was referred to our pediatric rheumatology department for suspected Still’s disease. He had a 4-month history of recurrent fever, intermittent knee pain (especially at the onset of gait), normocytic normochromic anemia (hemoglobin 8,8 g/dL) and sedimentation rate 120 mm/hour. An asymmetric genu varum was the only finding on physical examination.

This child had a previous story of congenital syphilis diagnosed and treated in the neonatal period. At this time he was abandoned by his mother, and then was institutionalized until 4 months when he was adopted. At the age of 8 months a painful right parasternal mass was noted. Histology revealed “nonspecific inflammatory reaction” and resolution occurred in one month. The Venereal Disease Research Laboratory (VDRL) test was 1:2 and *T. pallidum* hemagglutination (TPHA) test was reactive with a titer of 1:10.240. Posterior serological evaluations revealed a negative VDRL and declining of TPHA until 1:2560 in the second year of life. At 20 month-old he was referred to the orthopedics hospital department because of a bilateral genu varum. A “Blount’s disease” was diagnosed.

Given the past medical history of congenital syphilis, probably parasternal gumma at 8 month-old with TPHA titer of 10.240, penicillin treatment was started and maintained for 14 days. The treatment was successful and he became asymptomatic.

## Conclusion

The case reported highlights unusual evolution of congenital syphilis, despite conventional treatment of the disease in the neonatal period, the child presented clinical signs of infection later manifested as recurrent fever and severe anemia. Therefore, a past history of syphilis should always be valued.

Regarding the increase in the incidence of syphilis, all pediatricians should be aware of the diverse clinical features of syphilis to enable early diagnosis of the disease. Moreover, regular follow-up is extremely important to evaluate treatment effectiveness.

## Disclosure of interest

None declared.

